# HAIR-AN syndrome: a systematic review of clinical, metabolic, and hormonal features in women of reproductive age

**DOI:** 10.61622/rbgo/2026rbgo60

**Published:** 2026-08-20

**Authors:** Luís Jesuíno de Oliveira Andrade, Gabriela Correia Matos de Oliveira, Luís Matos de Oliveira, Ainoan dos Santos Cajado, Hellen Rebeca Galvão dos Santos, Alcina Maria Vinhaes Bittencourt, Osmário Jorge de Mattos Salles

**Affiliations:** 1 Universidade Estadual de Santa Cruz Departamento de Saúde Ilhéus BA Brazil Departamento de Saúde, Universidade Estadual de Santa Cruz, Ilhéus, BA, Brazil.; 2 Hospital Electro Bonini e Maternidade Cidinha Bonini Ribeirão Preto SP Brazil Hospital Electro Bonini e Maternidade Cidinha Bonini, Ribeirão Preto, SP, Brazil.; 3 Universidade Federal da Bahia Faculdade de Medicina Salvador BA Brazil Faculdade de Medicina, Universidade Federal da Bahia, Salvador, BA, Brazil.; 4 Escola Bahiana de Medicina e Saúde Pública Salvador BA Brazil Escola Bahiana de Medicina e Saúde Pública, Salvador, BA, Brazil.

**Keywords:** HAIR-AN syndrome, Hyperandrogenism, Insulin resistance, Acanthosis nigricans

## Abstract

**Objective:**

To synthesize the current evidence on the clinical, metabolic, and hormonal characteristics of HAIR-AN syndrome in women of reproductive age, and to evaluate current treatment options.

**Methods:**

A comprehensive literature search was conducted using PubMed, Embase, Scopus, and Web of Science databases up to June 2025. Eligible studies included observational and interventional designs reporting on women diagnosed with HAIR-AN syndrome. Data extraction focused on anthropometric, hormonal, and metabolic parameters. Risk of bias and methodological quality were assessed using the Newcastle-Ottawa Scale for observational studies and the Cochrane Risk of Bias tool for interventional studies.

**Results:**

From an initial pool of 226 records, five studies met inclusion criteria. Findings indicate that HAIR-AN syndrome predominantly affects obese women, with marked insulin resistance, hyperandrogenemia, and acanthosis nigricans severity correlating with metabolic dysfunction. Treatment strategies involving insulin-sensitizing agents, particularly incretin and metformin, demonstrated improvements in hormonal and metabolic profiles.

**Conclusion:**

HAIR-AN syndrome represents a unique and clinically relevant phenotype of severe IR with significant reproductive and dermatological manifestations. Early diagnosis and multidisciplinary management are essential to attenuate long-term metabolic complications.

**PROSPERO registry:** # CRD420251074124

## Introduction

Hyperandrogenism, Insulin Resistance, and Acanthosis Nigricans (HAIR-AN) syndrome is a rare but clinically significant metabolic-endocrine disorder primarily affecting women of reproductive age. This triad of clinical manifestations reflects an intricate interplay between hormonal dysregulation and insulin signaling pathways.^(1)^ Hyperandrogenism manifests with symptoms such as hirsutism and acne (menstrual irregularities commonly occur due to anovulation secondary to hyperandrogenism); insulin resistance (IR) predisposes individuals to type 2 diabetes mellitus (T2DM) and metabolic syndrome (MS); and acanthosis nigricans (AN) serves as a cutaneous marker of underlying IR.^(2)^ Although HAIR-AN has been increasingly recognized in the context of obesity and polycystic ovary syndrome (PCOS), it remains a distinct entity that warrants specific diagnostic and therapeutic attention due to its unique pathophysiological features and long-term complications.^(3)^

The three cardinal components of HAIR-AN syndrome - hyperandrogenism, IR, and AN - are interconnected through shared molecular mechanisms. Hyperandrogenism may originate from both ovarian and adrenal sources, driven by increased luteinizing hormone (LH) secretion and enhanced adrenal androgen production, often exacerbated by hyperinsulinemia.^(4)^ IR, particularly in skeletal muscle and adipose tissue, leads to compensatory hyperinsulinemia, which further aggravates androgen synthesis and decreases sex hormone-binding globulin (SHBG) levels.^(5)^ AN, characterized by velvety, hyperpigmented skin patches typically located in flexural areas, is considered a visible manifestation of insulin receptor dysfunction, likely mediated by insulin-like growth factor-1 receptor (IGF-1R) activation in keratinocytes and fibroblasts.^(6)^

While HAIR-AN syndrome shares some pathophysiological features with PCOS, including hyperinsulinemia and hyperandrogenism, it is distinguished by the presence of severe insulin resistance with acanthosis nigricans as a pathognomonic cutaneous marker, more pronounced adipose tissue dysfunction (‘adiposopathy’), and typically more severe metabolic derangement. Unlike PCOS, which represents a spectrum of phenotypes, HAIR-AN syndrome consistently presents with the complete triad of hyperandrogenism, profound insulin resistance, and acanthosis nigricans, warranting distinct diagnostic and therapeutic approaches.

Despite growing awareness of HAIR-AN syndrome, there remains a notable gap in the literature regarding its epidemiology, natural history, and optimal management strategies. Most studies report small case series or cross-sectional analyses, limiting generalizability and causal inference. Furthermore, there is heterogeneity in diagnostic criteria across studies, which complicates comparative analyses and the establishment of standardized treatment protocols. Additionally, while HAIR-AN is frequently discussed in the context of PCOS, few investigations have specifically examined this syndrome as a discrete clinical entity, leaving critical questions about its etiology, progression, and response to therapy unanswered.

Systematic reviews offer a robust methodological framework for synthesizing existing evidence, identifying patterns across studies, and quantifying effect sizes in heterogeneous populations.^(7)^ In the context of HAIR-AN syndrome, such an approach would allow for a comprehensive evaluation of the prevalence, associated comorbidities, and treatment outcomes across different demographic and clinical subgroups. By pooling data from diverse observational and interventional studies, a systematic review can enhance statistical power, reduce bias, and provide more reliable estimates than individual studies alone.^(8)^

Therefore, the objective of this systematic review is to comprehensively synthesize the extant literature on HAIR-AN syndrome, emphasizing its clinical manifestations, underlying pathophysiological mechanisms, and the spectrum of associated metabolic and endocrine abnormalities, as well as critically appraising the efficacy and safety of current therapeutic modalities, with the ultimate aim of informing clinical practice and guiding future research directions in the management of this complex syndrome.

## Methods

This systematic review was conducted in accordance with the Preferred Reporting Items for Systematic Reviews and Meta-Analyses (PRISMA) guidelines to ensure transparency, rigor, and reproducibility.^(9)^ The protocol was registered in advance on PROSPERO (registration number: CRD420251074124), aligning with international standards for evidence synthesis.

### Literature search strategy

A comprehensive literature search was performed using major electronic databases including PubMed, Embase, Scopus, and Web of Science, up to June 2025. Keywords and Medical Subject Headings (MeSH terms) related to "HAIR-AN syndrome", "hyperandrogenism", "insulin resistance", "acanthosis nigricans", and "polycystic ovary syndrome" were combined using Boolean operators. Reference lists of eligible articles and relevant reviews were manually screened to identify additional studies not captured by the electronic search.

### Inclusion and exclusion criteria

Studies were included if they: reported original data on women diagnosed with HAIR-AN syndrome; focused on clinical, metabolic, or hormonal parameters; and were published in peer-reviewed journals in English. Case reports, editorials, commentaries, and studies involving mixed populations without specific data on HAIR-AN patients were excluded.

### Study selection and data extraction

Two independent reviewers (LJ and LM) screened titles and abstracts for relevance. Full texts of potentially eligible studies were retrieved and assessed for inclusion. When full-text articles were unavailable through institutional access or direct author contact, these studies were excluded. Disagreements were resolved through discussion or consultation with a third reviewer. No studies were excluded due to unavailability of full-text articles. A standardized data extraction form was used to collect information on study characteristics (design, sample size, country of origin), demographic variables (age, ethnicity), anthropometric measurements (body mass index, waist circumference, waist-to-hip ratio), metabolic parameters (fasting glucose, fasting insulin, HOMA-IR index, HbA1c, lipid profile), and hormonal parameters (total testosterone, free testosterone, sex hormone-binding globulin, luteinizing hormone, follicle-stimulating hormone, dehydroepiandrosterone sulfate, 17-hydroxyprogesterone).

### Quality assessment

The methodological quality of included studies was assessed using the Newcastle-Ottawa Scale (NOS) for observational studies and the Cochrane Risk of Bias 2 (RoB 2) tool for interventional studies. For NOS, studies were rated based on selection, comparability, and outcome assessment criteria. For RoB 2, domains including randomization process, deviations from intended interventions, missing outcome data, measurement of outcomes, and selection of reported results were evaluated. Only observational studies scoring ≥5 stars on the NOS were considered for final synthesis to ensure robustness of findings.

### Statistical considerations

In instances where hazard ratios or survival data had been available, further statistical modeling would have been pursued. However, due to the cross-sectional nature of most included studies and absence of longitudinal follow-up data, such analyses were beyond the scope of this review. Descriptive synthesis was employed to summarize key clinical and biochemical features across studies. Where feasible, pooled estimates of mean values and standard deviations were calculated for continuous outcomes such as BMI, fasting insulin, glucose, HOMA-IR, and testosterone levels. Given the heterogeneity in study designs and populations, formal meta-analysis was not performed. Instead, qualitative synthesis was prioritized to reflect the diversity and consistency of reported outcomes. The inclusion and exclusion criteria, along with the rigorous selection process, were systematically documented in accordance with the PRISMA 2020 guidelines to ensure transparency and reproducibility throughout the stages of study identification, screening, eligibility assessment, and final inclusion.

## Results

The results of the search strategy are summarized in a PRISMA flow diagram (Figure 1), which illustrates the sequential process of study selection for inclusion in the review.

**Figure 1:**
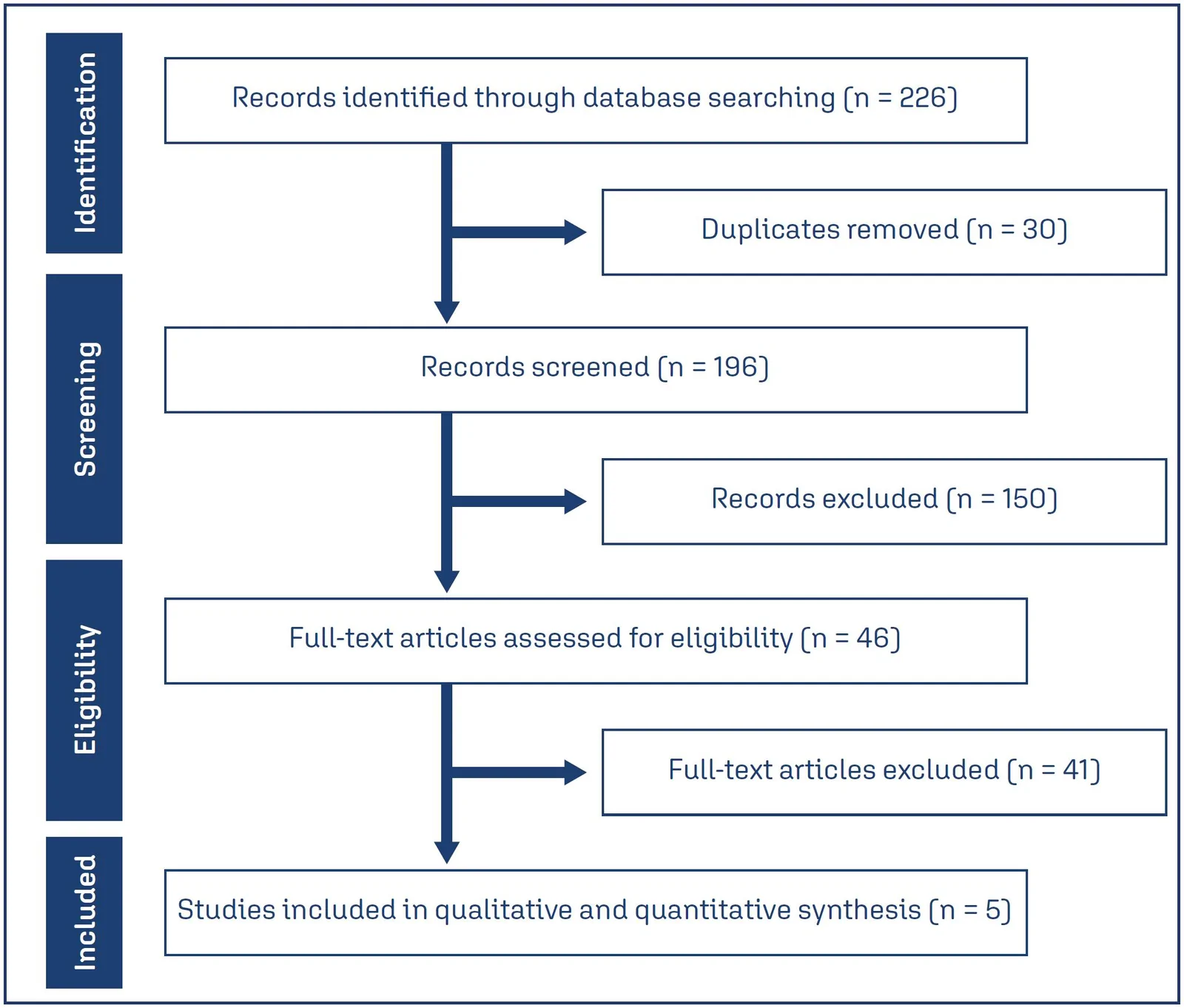
PRISMA studies selection flow diagram

### Systematic review results

The PRISMA flow diagram illustrates the rigorous selection process for studies included in this systematic review. Out of 226 records initially identified through database searching (PubMed: 78 records; Embase: 64 records; Scopus: 51 records; Web of Science: 33 records), 30 duplicates were removed, resulting in 196 records screened by title and abstract. Following this, 150 records were excluded based on relevance and eligibility criteria, leaving 46 full-text articles for detailed assessment. Of these, 41 articles were excluded after full-text review for not meeting the inclusion criteria, resulting in a final inclusion of 5 studies in the qualitative and quantitative synthesis. This transparent reporting ensures methodological rigor and reproducibility in accordance with PRISMA 2020 guidelines.^(9)^

### Characteristics of the included studies

The five included studies comprised three cross-sectional studies (Agrawal et al., 2021; Dunaif et al., 1991; Flier et al., 1985), one case series (Livadas et al., 2020), and one observational cohort study (Barth et al., 1988). No longitudinal follow-up data were available beyond the index assessment in any of the included studies.

- Agrawal K et al. (2021)^(10)^: This observational study compared HAIR-AN syndrome with PCOS in Asian Indian girls, underscoring the distinct pathophysiology of HAIR-AN as primarily driven by adipose tissue dysfunction ("adiposopathy"). Women with HAIR-AN exhibited significantly higher IR indices, hyperinsulinemia, and obesity compared to PCOS controls. The study also documented a more severe metabolic profile, including dyslipidemia and impaired glucose tolerance. These findings suggest that HAIR-AN represents an extreme phenotype within the spectrum of IR disorders, with adipose tissue dysfunction playing a central role.
- Dunaif A et al. (1991)^(11)^: This investigation focused on the biochemical and clinical characterization of AN in hyperandrogenic women. The authors found that clinical skin examination was relatively insensitive for detecting AN, suggesting that histological confirmation may be necessary in ambiguous cases. Biochemically, the study demonstrated that IR correlated more robustly with hyperinsulinemia than with androgen levels, indicating that IR is a primary defect. The study also highlighted the heterogeneity of insulin receptor sensitivity among affected individuals (Chart Supplementary).
- Livadas et al. (2020)^(12)^: This study evaluated the therapeutic impact of liraglutide on metabolic and reproductive parameters in women diagnosed with HAIR-AN syndrome. The findings revealed that liraglutide significantly improved insulin sensitivity, reduced hyperandrogenemia, and ameliorated menstrual irregularities, suggesting a beneficial role in both metabolic and endocrine regulation. Additionally, reductions in body mass index and visceral adiposity were observed, highlighting its potential as an adjunct or alternative therapy in the management of HAIR-AN syndrome. The results suggest a potential role for Glucagon-like peptide-1 (GLP-1) receptor agonists in the management of patients with HAIR-AN syndrome, although larger randomized controlled trials are necessary to establish efficacy and support clinical integration into treatment protocols.
- Barth JH, et al. (1988)^(13)^: This article explores the interplay between AN, IR, and cutaneous virilism in hyperandrogenic females. It underscores the utility of dermatological manifestations as clinical markers of underlying metabolic dysfunction, particularly in the context of insulin signaling abnormalities. The authors demonstrate that the severity of AN correlates with the degree of IR rather than androgen excess per se, reinforcing its role as a cutaneous indicator of metabolic dysregulation. Furthermore, the presence of cutaneous virilism was associated with increased adrenal androgen production, suggesting a multifactorial pathogenesis requiring integrated diagnostic and therapeutic approaches.
- Flier JS, et al. (1985)^(14)^: describe a distinct metabolic phenotype characterized by the triad of AN, hyperandrogenism, and IR in obese women, delineating it from the classical syndromic forms of IR such as type A (genetic severe IR with hyperandrogenism) and type B (autoimmune insulin receptor dysfunction). The study identifies AN as a consistent cutaneous marker of profound IR in this cohort, independent of circulating androgen levels.

The characteristics of the included studies are presented in chart 1. Such comprehensive tabulation facilitates a clear understanding of study heterogeneity and supports the contextual interpretation of the meta-analytic findings. The inclusion of this detailed summary ensures transparency and aids reproducibility in accordance with best practices for systematic reviews.

**Chart 1:** Overview of the included studies

Study	Design	Sample (n)	Age (mean ± SD)	Mean BMI (kg/m^2)^	Mean fasting insulin	Fasting glucose	HOMA-IR	Testosterone	AN prevalence
Agrawal, et al. 2021^(10)^	Cross-sectional	30	21.8 ± 4.8	28.3 ± 2.5	37.8 ± 3.4 µIU/ml	90.2 ± 7.8 mg/dL	8.4 ± 1.2	83.9 ± 7.4 ng/dL	100%
Dunalf A, et al. 1991^(11)^	Observational	19	25.0 ± 3.5	37.2 ± 6.5	316.0 ± 172.0 pmol/L	4.9 ± 0.3 mmol/L	11.2 ± 3.2	0.8 ± 0.4 mmol/L	100%
Livadas, et al. 2020^(12)^	Case series	5	29.0 ± 2.4	31.0 ± 3.5	39.3 ± 4.8 pmoL/L	85.3 ± 5.2 mg/dL	8.4 ± 1.3	1.0 ± 0.1 ng/dL	100%
Barth JG, et al. 1988 ^(13)^	Case/control	13	35.0 ± 5.4	34.6 ± 3.8	33.0 ± 2.5 µIU/ml	5.1 ± 0.5 mmol/L	8.0 ±1.2	2.7 ± 0.9 mmol/L	100%
Flier JS, et al. 1985^(14)^	Observational	7	21.0 ± 2.0	39.4 ± 9.9	58.0 ± 4.0 µIU/ml	92.0 ± 4.0 mg/dL	13.6 ± 3.9	1.0 ± 0.2 ng/dL	100%

### Risk of bias and methodological quality assessment of included studies

The overall methodological quality of the included studies ranged from moderate to high, with the majority demonstrating adequate selection and outcome assessment but variable comparability (Chart 2).

**Chart 2:** Assessment of risk of bias and methodological quality of included studies

Study	Selection (max 4)	Comparability (max 3)	Outcome/Exposure (max 3)	Total NOS Score (max 9)	Quality rating
Agrawal, et al. 2021^(10)^	****	**	***	8	Moderate
Dunalf A, et al. 1991^(11)^	***	**	**	7	Moderate
Livadas, et al. 2020^(12)^	**	*	*	5	Low
Barth JG, et al. 1988^(13)^	***	**	**	7	Moderate
Flier JS, et al. 1985^(14)^	***	**	**	7	Moderate

The risk of bias was lowest in studies with robust cohort designs and explicit adjustment for confounders, and highest in case series or studies with limited reporting of outcomes. Application of the NOS facilitated a transparent and systematic appraisal of study quality, supporting the validity of the synthesized evidence while highlighting areas requiring further research and methodological rigor. Based on the comprehensive analysis of the selected studies, an algorithm for the diagnosis of HAIR-AN syndrome has been developed, integrating key clinical, biochemical, and histopathological parameters (Figure 2). The algorithm prioritizes the identification of AN as a consistent dermatological hallmark, alongside objective assessment of IR indices and hyperinsulinemia, as emphasized by Agrawal et al.^(10)^ and Dunaif et al.^(11)^ It incorporates differentiation from phenotypically similar disorders such as PCOS and classical IR syndromes (types A and B), as delineated by Flier et al.^(14)^ Furthermore, the algorithm underscores the necessity of evaluating hyperandrogenism through comprehensive hormonal profiling, including adrenal and ovarian androgen sources, acknowledging the multifactorial pathogenesis highlighted by Barth et al.^(13)^ Therapeutic responsiveness, particularly to agents such as incretin demonstrated by Livadas et al.,^(12)^ is considered in refining diagnostic certainty and guiding management. This structured approach facilitates early and accurate diagnosis, enabling targeted interventions tailored to the complex metabolic-endocrine phenotype characteristic of HAIR-AN syndrome (Supplementary material – Chart 1).

**Figure 2:**
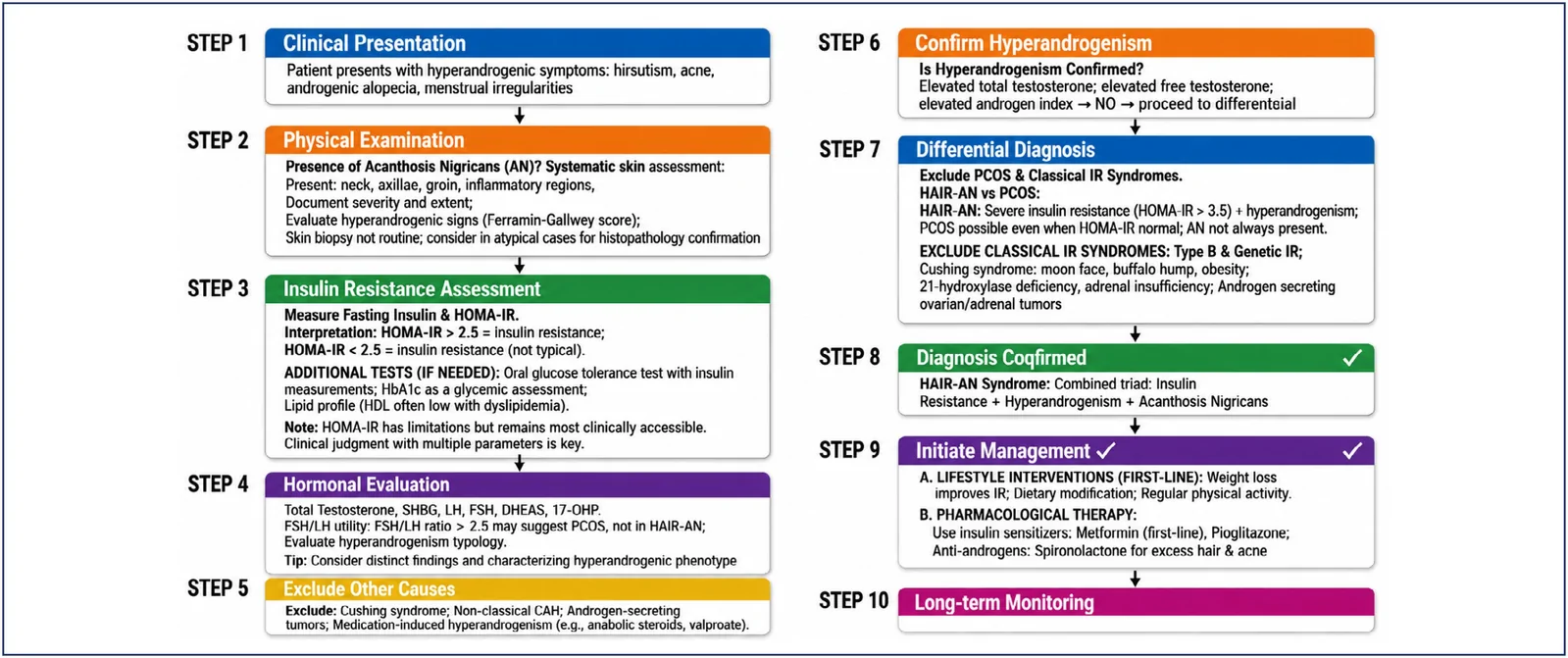
Algorithm for the diagnosis of HAIR-AN syndrome

**Source:** Flowchart developed by the authors based on synthesis of included studies and current clinical guidelines.

## Discussion

The present systematic review provides a synthesis of the current evidence on HAIR-AN syndrome, highlighting its distinct clinical, metabolic, and hormonal profile among women of reproductive age. By delineating the interplay between hyperandrogenism, severe IR, and AN, this review underscores the importance of recognizing HAIR-AN as a unique endocrinopathy rather than a mere variant of PCOS. The findings reinforce the role of adipose tissue dysfunction and post-receptor insulin signaling defects as central pathophysiological mechanisms driving the syndrome's phenotypic expression. While adipose tissue dysfunction and post-receptor insulin signaling defects are also features of PCOS, HAIR-AN syndrome is distinguished by the severity and obligate presence of these abnormalities. In HAIR-AN, the degree of adipose tissue dysfunction (‘adiposopathy’ or ‘sick fat’) is more profound, leading to more severe compensatory hyperinsulinemia and consequently more pronounced acanthosis nigricans. Additionally, HAIR-AN demonstrates more consistent and severe metabolic derangement, including higher HOMA-IR values, greater dyslipidemia, and earlier progression to glucose intolerance compared to typical PCOS phenotypes.

Clinically, the presence of AN emerges as a key cutaneous biomarker of underlying metabolic dysregulation, warranting targeted screening and early intervention. These insights contribute to improved diagnostic accuracy and the development of tailored therapeutic strategies aimed at attenuate both reproductive and metabolic morbidity associated with HAIR-AN syndrome.

HAIR-AN syndrome emerges as a clinically relevant yet frequently overlooked condition within the spectrum of IR and hyperandrogenic disorders.^(15)^ It often manifests during childhood or early adolescence with signs of IR, frequently accompanied by obesity, which may precede the onset of other clinical features. This syndrome is estimated to affect approximately 5% of women presenting with hyperandrogenic symptoms, although it remains underdiagnosed due to its overlap with more common conditions such as PCOS.^(3)^ Accumulating evidence underscores the role of intrinsic adipose tissue dysfunction in driving compensatory hyperinsulinemia and subsequent ovarian hyperandrogenism.^(16)^ The presence of AN serves not only as a dermatologic marker but also as a sentinel sign of underlying metabolic derangement.^(17)^ Accurate identification of HAIR-AN syndrome is fundamental, as it carries an increased risk of developing T2DM and MS, necessitating early intervention and long-term monitoring.^(18)^ Our systematic review corroborates the important role of adipose tissue dysfunction and hyperinsulinemia as central drivers of the HAIR-AN syndrome phenotype, delineating a distinct metabolic-endocrine profile that differentiates it from related hyperandrogenic disorders. The included studies consistently highlight AN as a reliable clinical biomarker of severe IR and metabolic derangement.

In young women, hyperandrogenism typically presents with a constellation of dermatologic and endocrine manifestations, including seborrhea, excessive terminal hair growth, persistent acne, and menstrual cycle disturbances. In more severe or advanced cases, patients may exhibit androgenic alopecia, vocal deepening, clitoral hypertrophy, and alterations in muscle mass composition, reflecting the systemic impact of androgen excess.^(19,20)^ IR, a hallmark of HAIR-AN syndrome, exhibits heterogeneous clinical phenotypes; some individuals demonstrate marked hyperinsulinemia despite maintaining normoglycemia, whereas others progress to overt hyperglycemia within diabetic thresholds.^(21,22)^ This variability underscores the complexity of metabolic dysregulation inherent to the syndrome and necessitates comprehensive biochemical evaluation for accurate diagnosis and management. Our study consistently identified IR as a universal feature across all included studies, underscoring its central role in the pathophysiology of HAIR-AN syndrome. Similarly, elevated total testosterone levels were uniformly reported, reflecting the persistent hyperandrogenic state characteristic of this condition. These findings reinforce the intertwined metabolic and endocrine disturbances fundamental to the syndrome's clinical phenotype.

AN in HAIR-AN syndrome represents a cutaneous manifestation of severe IR, characterized by hyperkeratotic, velvety, hyperpigmented plaques predominantly affecting flexural areas.^(6)^ The pathophysiology involves hyperinsulinemia-induced activation of insulin-like growth factor-1 (IGF-1) receptors on keratinocytes and dermal fibroblasts, promoting aberrant cellular proliferation and epidermal hyperplasia.^(23)^ AN functions as an epiphenomenon of underlying metabolic dysfunction, with lesion severity correlating directly with IR severity.^(24)^ The histopathological features include papillomatosis, acanthosis, and hyperkeratosis, reflecting the dysregulated IGF-1 signaling pathways characteristic of this severe MS.^(25)^ All studies included in our systematic review consistently reported the presence of acanthosis nigricans in 100% of their respective participant cohorts, underscoring its universal manifestation within HAIR-AN syndrome. This uniform dermatological finding reinforces the critical role of severe insulin resistance and hyperinsulinemia-driven epidermal proliferation as pathognomonic features of the syndrome. Thus, the consistent identification of acanthosis nigricans across diverse populations further validates its utility as a reliable clinical biomarker for early diagnosis and risk stratification in affected individuals.

The laboratory diagnosis of HAIR-AN syndrome requires a comprehensive biochemical evaluation to establish the characteristic triad of severe IR, hyperandrogenemia, and metabolic dysfunction.^(26)^ Essential laboratory investigations include measurement of fasting glucose and insulin levels to calculate the homeostatic model assessment of IR, with values typically exceeding 2.5 indicating significant IR.^(27)^ Quantification of serum total testosterone is imperative to confirm hyperandrogenic status, while measurement of sex hormone-binding globulin (SHBG) facilitates calculation of the free androgen index, enhancing diagnostic accuracy. A detailed hormonal panel comprising luteinizing hormone (LH), follicle-stimulating hormone (FSH), dehydroepiandrosterone sulfate (DHEAS), and 17-hydroxyprogesterone (17-OHP) is essential to exclude alternative etiologies such as congenital adrenal hyperplasia and other androgen excess disorders.^(28)^ Additional metabolic parameters include lipid profile assessment, as patients demonstrate increased risk for dyslipidemia and cardiovascular complications.^(29)^ The integration of these laboratory findings with clinical manifestations enables accurate diagnosis and appropriate therapeutic intervention. In our systematic review, four of the five included studies comprehensively assessed metabolic profiles and adrenal-ovarian hormonal axes, including SHBG, LH, FSH, DHEAS, and 17-OHP, to exclude confounding etiologies. However, one study omitted these parameters, limiting its diagnostic precision and the comprehensive characterization of the hormonal phenotype. However, total testosterone quantification was uniformly performed across all studies, confirming hyperandrogenemia as a universal diagnostic criterion in HAIR-AN syndrome.

Current treatment of HAIR-AN syndrome involves a multifaceted approach prioritizing lifestyle modification and weight reduction to improve insulin sensitivity. Pharmacotherapy includes insulin sensitizers such as metformin and pioglitazone, which reduce hyperinsulinemia and androgen levels.^(30,31)^ Antiandrogens like spironolactone and flutamide alleviate hirsutism, often combined with estrogen-progestin oral contraceptives to suppress ovarian androgen production.^(32)^ Emerging therapies include GLP-1 receptor agonists, which improve metabolic and reproductive parameters independently of weight loss.^(33,34)^ In refractory or severe obesity cases, bariatric surgery, particularly sleeve gastrectomy, has demonstrated significant and sustained normalization of insulin resistance and hyperandrogenism in women with PCOS.^(35-37)^ While direct evidence in HAIR-AN syndrome is limited, given the shared pathophysiology of severe insulin resistance and hyperandrogenism, bariatric surgery may represent a potential therapeutic option for carefully selected patients with HAIR-AN and severe obesity refractory to medical management. However, prospective studies specifically in HAIR-AN populations are needed to establish efficacy and safety. Multidisciplinary care addressing psychological aspects is essential for treatment adherence and outcomes.^(38)^ Based on our study, summarized in Table 1, therapeutic strategies for HAIR-AN syndrome indicate that the combination of lifestyle modifications and pharmacological interventions, particularly metformin and antiandrogens, consistently improves metabolic and androgenic parameters. Specifically, GLP-1 receptor agonists and bariatric surgery have shown promising results in refractory cases, leading to significant reductions in insulin resistance and the restoration of menstrual regularity.

## Conclusion

HAIR-AN syndrome represents a unique and clinically relevant metabolic-endocrine disorder characterized by severe IR with significant reproductive, dermatological, and hormonal manifestations in women of reproductive age. Early recognition and multidisciplinary, tailored interventions are fundamental to preventing long-term metabolic complications and improving reproductive outcomes in affected women.

## Data Availability

The research data are described in the article presented.

## References

[1] O’Brien B, Dahiya R, Kimble R (2020). Hyperandrogenism, insulin resistance and acanthosis nigricans (HAIR-AN syndrome): an extreme subphenotype of polycystic ovary syndrome. BMJ Case Rep.

[2] Zeng X, Xie YJ, Liu YT, Long SL, Mo ZC (2020). Polycystic ovarian syndrome: correlation between hyperandrogenism, insulin resistance and obesity. Clin Chim Acta.

[3] Gheshlagh RA, Topsakal S (2025). A Complex Clinical Situation in Polycystic Ovary Syndrome: HAIR-AN Syndrome "Case Report". Case Rep Med.

[4] Barbieri RL, Ryan KJ (1983). Hyperandrogenism, insulin resistance, and acanthosis nigricans syndrome: a common endocrinopathy with distinct pathophysiologic features. Am J Obstet Gynecol.

[5] Petersen MC, Shulman GI (2018). Mechanisms of insulin action and insulin resistance. Physiol Rev.

[6] Leung AK, Lam JM, Barankin B, Leong KF, Hon KL (2022). Acanthosis nigricans: an updated review. Curr Pediatr Rev.

[7] Scheidt S, Vavken P, Jacobs C, Koob S, Cucchi D, Kaup E (2019). Systematic reviews and meta-analyses. Z Orthop Unfall.

[8] Lamberink HJ, Otte WM, Sinke MR, Lakens D, Glasziou PP, Tijdink JK (2018). Statistical power of clinical trials increased while effect size remained stable: an empirical analysis of 136,212 clinical trials between 1975 and 2014. J Clin Epidemiol.

[9] Page MJ, McKenzie JE, Bossuyt PM, Boutron I, Hoffmann TC, Mulrow CD (2021). The PRISMA 2020 statement: an updated guideline for reporting systematic reviews. BMJ.

[10] Agrawal K, Mathur R, Purwar N, Mathur SK, Mathur DK (2021). Hyperandrogenism, Insulin Resistance, and Acanthosis Nigricans (HAIR-AN) syndrome reflects adipose tissue dysfunction ("Adiposopathy" or "Sick Fat") in Asian Indian girls. Dermatology.

[11] Dunaif A, Green G, Phelps RG, Lebwohl M, Futterweit W, Lewy L Acanthosis Nigricans, insulin action, and hyperandrogenism: clinical, histological, and biochemical findings. J Clin Endocrinol Metab.

[12] Livadas S, Androulakis I, Angelopoulos N, Lytras A, Papagiannopoulos F, Kassi G (2020). Liraglutide administration improves hormonal/metabolic profile and reproductive features in women with HAIR-AN syndrome. Endocrinol Diabetes Metab Case Rep.

[13] Barth JH, Ng LL, Wojnarowska F, Dawber RP (1988). Acanthosis nigricans, insulin resistance and cutaneous virilism. Br J Dermatol.

[14] Flier JS, Eastman RC, Minaker KL, Matteson D, Rowe JW (1985). Acanthosis nigricans in obese women with hyperandrogenism. Characterization of an insulin-resistant state distinct from the type A and B syndromes. Diabetes.

[15] Azziz R, Carmina E, Chen Z, Dunaif A, Laven JS, Legro RS (2016). Polycystic ovary syndrome. Nat Rev Dis Primers.

[16] Barbieri RL, Smith S, Ryan KJ (1988). The role of hyperinsulinemia in the pathogenesis of ovarian hyperandrogenism. Fertil Steril.

[17] Radu AM, Carsote M, Dumitrascu MC, Sandru F (2022). Acanthosis Nigricans: Pointer of Endocrine Entities. Diagnostics (Basel).

[18] Unluhizarci K, Karaca Z, Kelestimur F (2021). Role of insulin and insulin resistance in androgen excess disorders. World J Diabetes.

[19] Sardana K, Muddebihal A, Sehrawat M, Bansal P, Khurana A (2024). An updated clinico-investigative approach to diagnosis of cutaneous hyperandrogenism in relation to adult female acne, female pattern alopecia & hirsutism a primer for dermatologists. Expert Rev Endocrinol Metab.

[20] Witchel SF, Azziz R, Oberfield SE (2022). History of polycystic ovary syndrome, premature adrenarche, and hyperandrogenism in pediatric endocrinology. Horm Res Paediatr.

[21] Kastury R, Joseph K, Caprio S, Boulware S, Ma D, Vash-Margita A (2022). Genetic heterogeneity in the hair-an sub-phenotype of PCOS. J Pediatr Adolesc Gynecol.

[22] Szablewski L (2024). Changes in cells associated with insulin resistance. Int J Mol Sci.

[23] Banti S, Sumathy TK, Pramila K (2022). Insulin resistance in various grades of acanthosis nigricans. Acta Dermatovenerol Alp Panonica Adriat.

[24] Burguete-García AI, Ramírez Valverde AG, Espinoza-León M, Vázquez IS, Estrada Ramírez EY, Maldonado-López I (2022). Severe quantitative scale of acanthosis nigricans in neck is associated with abdominal obesity, HOMA-IR, and hyperlipidemia in obese children from Mexico city: A Cross-Sectional Study. Dermatol Res Pract.

[25] Mohaghegh F, Shoushtarizadeh M, Bahraminejad M, Tabatabaei ET, Talebzadeh Z (2025). Presentation of acanthosis nigricans as scalp papillomatosis: a case report. J Med Case Rep.

[26] Lewin Z, Vitek WS, O’Malley W, Astapova O (2023). Resolution of Hyperandrogenism, Insulin Resistance and Acanthosis Nigricans (HAIR-AN) syndrome after sleeve gastrectomy. JCEM Case Rep.

[27] Tang Q, Li X, Song P, Xu L (2015). Optimal cut-off values for the homeostasis model assessment of insulin resistance (HOMA-IR) and pre-diabetes screening: developments in research and prospects for the future. Drug Discov Ther.

[28] Azziz R, Amiri M, Bril F, Joham AE, Kelestimur F, Ottey S (2025). Approach to the Patient: hirsutism. J Clin Endocrinol Metab.

[29] Yu J, Zhou Y, Ding J, Zhang D, Yu C, Huang H (2023). Characteristics and possible mechanisms of metabolic disorder in overweight women with polycystic ovary syndrome. Front Endocrinol (Lausanne).

[30] Ali DE, Shah M, Ali A, Malik MO, Rehman F, Badshah H (2019). Treatment with Metformin and Combination of Metformin Plus Pioglitazone on Serum Levels of IL-6 and IL-8 in Polycystic Ovary Syndrome: A Randomized Clinical Trial. Horm Metab Res.

[31] Jianfang F, Wanxia X, Xiling G, Jing X, Wenjuan Y, Jianrong L (2023). Effect and safety of pioglitazone-metformin tablets in the treatment of newly diagnosed type 2 diabetes patients with nonalcoholic fatty liver disease in shaanxi province: a randomized, double-blinded, double-simulated multicenter study. J Diabetes Res.

[32] Karakurt F, Sahin I, Güler S, Demirbas B, Culha C, Serter R (2008). Comparison of the clinical efficacy of flutamide and spironolactone plus ethinyloestradiol/cyproterone acetate in the treatment of hirsutism: a randomised controlled study. Adv Ther.

[33] Zhou L, Qu H, Yang L, Shou L (2023). Effects of GLP1RAs on pregnancy rate and menstrual cyclicity in women with polycystic ovary syndrome: a meta-analysis and systematic review. BMC Endocr Disord.

[34] Austregésilo de Athayde De Hollanda Morais B, Martins Prizão V, de Moura de Souza M, Ximenes Mendes B, Rodrigues Defante ML, Cosendey Martins O (2024). The efficacy and safety of GLP-1 agonists in PCOS women living with obesity in promoting weight loss and hormonal regulation: A meta-analysis of randomized controlled trials. J Diabetes Complications.

[35] Lee R, Joy Mathew C, Jose MT, Elshaikh AO, Shah L, Cancarevic I (2020). A review of the impact of bariatric surgery in women with polycystic ovary syndrome. Cureus.

[36] Skubleny D, Switzer NJ, Gill RS, Dykstra M, Shi X, Sagle MA (2016). The impact of bariatric surgery on polycystic ovary syndrome: a systematic review and meta-analysis. Obes Surg.

[37] Samarasinghe SN, Leca B, Alabdulkader S, Dimitriadis GK, Davasgaium A, Thadani P (2024). Bariatric surgery for spontaneous ovulation in women living with polycystic ovary syndrome: the BAMBINI multicentre, open-label, randomised controlled trial. Lancet.

[38] Omar HA, Logsdon S, Richards J (2004). Clinical profiles, occurrence, and management of adolescent patients with HAIR-AN syndrome. ScientificWorldJournal.

