# Supplementary material for: HAIR-AN syndrome: a systematic review of clinical, metabolic, and hormonal features in women of reproductive age
Source: Rev Bras Ginecol Obstet. 2026 Aug 20;48:e-rbgo60. doi: 10.61622/rbgo/2026rbgo60 (PMC13690388; doi:10.61622/rbgo/2026rbgo60)
Supplement: Supplementary file 1 [file 1806-9339-rbgo-48-e-rbgo60-Suppl01.pdf]

Supplementary material

Chart 1. Summary of study designs, key findings, and clinical relevance of the five studies included in this review on HAIR-AN syndrome

Study	Search strategy	Study design	Key findings	Clinical relevance	Conclusion
Agrawal K, Mathur R, Purwar N, Mathur SK, Mathur DK. Hyperandrogenism, Insulin Resistance, and Acanthosis Nigricans (HAIR-AN) Syndrome Reflects Adipose Tissue Dysfunction ("Adiposopathy" or "Sick Fat") in Asian Indian Girls. Dermatology. 2021;237(5):797-805. doi: 10.1159/000512918	Databases: PubMed, Embase, Scopus, Web of Science (up to June 2025).<br><br>MeSH terms & keywords: "HAIR-AN syndrome", "hyperandrogenism", "insulin resistance", "acanthosis nigricans", "polycystic ovary syndrome" — combined with Boolean operators.<br><br>Manual screening of reference lists of eligible articles and relevant reviews.	Cross-sectional (observational)	1. Compared HAIR-AN syndrome with PCOS in Asian Indian girls.<br>2. Women with HAIR-AN showed significantly higher IR indices, hyperinsulinemia, and obesity vs. PCOS controls.<br>3. More severe metabolic profile documented: dyslipidemia and impaired glucose tolerance.<br>4. Adipose tissue dysfunction ("adiposopathy") identified as primary driver.	1. HAIR-AN represents an extreme phenotype within the IR disorders spectrum.<br>2. Distinct pathophysiology from PCOS, with adiposopathy playing a central role.<br>3. Supports the need for targeted metabolic interventions beyond standard PCOS management.	The significantly raised adipocytokines in HAIR-AN syndrome patients compared to PCOS patients indicates the primary role of adipose tissue dysfunction ("adiposopathy") in the pathogenesis of HAIR-AN syndrome, while only a minor role, if any, in PCOS. Both syndromes stand as distinct entities pathogenically with an overlapping phenotype.
Dunaif A, Green G, Phelps RG, Lebwohl M, Futterweit W, Lewy L. Acanthosis Nigricans, insulin action, and hyperandrogenism: clinical, histological, and bio chemical findings. J Clin Endocrinol Metab. 1991;73(3):590-5. doi: 10.1210/jcem-73-3-590	Databases: PubMed, Embase, Scopus, Web of Science (up to June 2025).<br><br>MeSH terms & keywords: "HAIR-AN syndrome", "hyperandrogenism", "insulin resistance", "acanthosis nigricans", "polycystic ovary syndrome" — combined with Boolean operators.<br><br>Manual screening of reference lists of eligible articles and relevant reviews.	Cross-sectional	1. Biochemical and clinical characterization of acanthosis nigricans (AN) in hyperandrogenic women.<br>2. Clinical skin examination showed low sensitivity for AN detection; histological confirmation may be necessary.<br>3. IR correlated more robustly with hyperinsulinemia than with androgen levels.	1. IR is a primary defect, not secondary to androgen excess.<br>2. Highlights heterogeneity of insulin receptor sensitivity among affected individuals.<br>3. Underscores the need for biochemical (not solely clinical) assessment of AN.	1) Clinical skin examination was very insensitive for detecting AN; 2) the best biochemical correlate of histological AN was decreased insulin action, rather than insulin or androgen levels per se; and 3) AN is a very common epiphenomenon of insulin resistance, and its clinical presence should not be used as a criterion for stratifying hyperandrogenic women.
Livadas S, Androulakis I, Angelopoulos N, Lytras A, Papagiannopoulos F, Kassi G. Liraglutide administration improves hormonal/metabolic profile and reproductive features in women with HAIR-AN syndrome. Endocrinol Diabetes Metab Case Rep. 2020;2020:19-0150. doi: 10.1530/EDM-19-0150	Databases: PubMed, Embase, Scopus, Web of Science (up to June 2025).<br><br>MeSH terms & keywords: "HAIR-AN syndrome", "hyperandrogenism", "insulin resistance", "acanthosis nigricans", "polycystic ovary syndrome" — combined with Boolean operators.<br><br>Manual screening of reference lists of eligible articles and relevant reviews.	Case series	1. Evaluated therapeutic impact of liraglutide in women with HAIR-AN syndrome.<br>2. Liraglutide significantly improved insulin sensitivity, reduced hyperandrogenemia, and ameliorated menstrual irregularities.<br>3. Reductions in BMI and visceral adiposity were observed.	1. GLP-1 receptor agonists may serve as adjunct or alternative therapy for HAIR-AN.<br>2. Benefits span metabolic and endocrine domains.<br>3. Larger randomized controlled trials are needed to establish efficacy and support clinical integration.	Liraglutide administration improves hormonal/metabolic profile and reproductive features in women with HAIR-AN syndrome.
Barth JH, Ng LL, Wojnarowska F, Dawber RP. Acanthosis nigricans, insulin resistance and cutaneous virilism. Br J Dermatol. 1988;118(5):613-9. doi: 10.1111/j.1365-2133.1988.tb02561.x	Databases: PubMed, Embase, Scopus, Web of Science (up to June 2025).<br><br>MeSH terms & keywords: "HAIR-AN syndrome", "hyperandrogenism", "insulin resistance", "acanthosis nigricans", "polycystic ovary syndrome" — combined with Boolean operators.<br><br>Manual screening of reference lists of eligible articles and relevant reviews.	Observational cohort	1. Explored interplay between AN, IR, and cutaneous virilism in hyperandrogenic females.<br>2. Severity of AN correlated with degree of IR rather than with androgen excess per se.<br>3. Cutaneous virilism was associated with increased adrenal androgen production.	1. AN serves as a cutaneous indicator of metabolic dysregulation, not merely androgen excess.<br>2. Multifactorial pathogenesis requiring integrated diagnostic and therapeutic approaches.<br>3. Dermatological manifestations are clinically useful markers of underlying metabolic dysfunction.	Treatment of acanthosis nigricans with anti-androgenic agents has been unsuccessful. AN severity reflects insulin resistance rather than androgen excess, reinforcing its role as a cutaneous indicator of metabolic dysregulation.
Flier JS, Eastman RC, Minaker KL, Matteson D, Rowe JW. Acanthosis nigricans in obese women with hyperandrogenism. Characterization of an insulin-resistant state distinct from the type A and B syndromes. Diabetes. 1985;34(2):101-7. doi: 10.2337/diab.34.2.101	Databases: PubMed, Embase, Scopus, Web of Science (up to June 2025).<br><br>MeSH terms & keywords: "HAIR-AN syndrome", "hyperandrogenism", "insulin resistance", "acanthosis nigricans", "polycystic ovary syndrome" — combined with Boolean operators.<br><br>Manual screening of reference lists of eligible articles and relevant reviews.	Cross-sectional	1. Described a distinct metabolic phenotype: AN + hyperandrogenism + IR in obese women.<br>2. Delineated from classical syndromic IR forms: Type A (genetic severe IR) and Type B (autoimmune IR).<br>3. AN identified as a consistent cutaneous marker of profound IR, independent of circulating androgen levels.	1. Established HAIR-AN as a distinct clinical entity separate from other IR syndromes.<br>2. AN as a marker of profound IR regardless of androgen levels reinforces its diagnostic value.<br>3. Supports a metabolic-first diagnostic framework for this condition.	Acanthosis nigricans is present in as many as 5% of women with clinically significant hyperandrogenism. These women, although not diabetic, have fairly marked insulin resistance.

AN: acanthosis nigricans; BMI: body mass index; GLP-1: glucagon-like peptide-1; HAIR-AN: hyperandrogenism, insulin resistance, and acanthosis nigricans; IR: insulin resistance; PCOS: polycystic ovary syndrome.
